# The Effects of Iloprost on Isthmin-1, Adropin and Trpc6 Immunoreactivity in an Experimental Lower Extremity Ischemia–Reperfusion Injury Model

**DOI:** 10.3390/biomedicines14081828

**Published:** 2026-08-14

**Authors:** İbrahim Murat Özgüler, Latif Üstünel, Tuncay Kuloğlu

**Affiliations:** 1 Department of Cardiovascular Surgery, Faculty of Medicine, Firat University, 23200 Elazig, Turkey; latifustunel@hotmail.com; 2 Department of Histology and Embryology, Faculty of Medicine, Firat University, 23200 Elazig, Turkey; tkuloglu@firat.edu.tr

**Keywords:** adropin, iloprost, ischemia/reperfusion, isthmin-1, TRPC6

## Abstract

***Background and Objectives***: Ischemia–reperfusion (IR) injury to skeletal muscle induces both structural and functional impairments, primarily attributed to increased production of reactive oxygen species and subsequent inflammation following reperfusion. Iloprost (ILO), a stable prostacyclin analog, exerts cytoprotective effects through reduction in oxidative stress as well as by improvement of microvascular function. The purpose of the present study was to investigate the influence of iloprost on Isthmin-1 (ISM-1), adropin and transient receptor potential canonical 6 (TRPC6) immunoreactivity in rat lower extremity muscles in an experimental IR model. ***Materials and Methods***: Thirty male Wistar albino rats, were divided into five groups, each with an equal number of rats; Control, Sham, ILO, IR, and IR + ILO. The ILO group received an intravenous infusion of 2 ng/kg/min ILO. After intracardiac blood was collected, muscle tissue samples were rapidly removed. Serum total oxidant status (TOS) and total antioxidant status (TAS) levels were measured. Histopathological examination of the collected muscle tissue samples was performed using hematoxylin-eosin staining, and immunohistochemical staining was used to assess the immunoreactivity of ISM-1, adropin, and TRPC6. ***Results***: The IR group showed increased serum total oxidant status levels and immunoreactivity for ISM-1, adropin, and TRPC6, while total antioxidant status levels decreased. Compared with the IR group, the IR + ILO group showed decreased serum TOS levels and immunoreactivity for ISM-1, adropin, and TRPC6, while TAS levels increased. ***Conclusions***: Treatment with ILO may have a protective effect on the skeletal muscle of lower extremities exposed to ischemia–reperfusion injury. This effect may be due to a reduction in oxidative stress and the regulation of immunoreactivity of ISM-1, adropin, and TRPC6.

## 1. Introduction

Tissue and organ ischemia is among the leading causes of morbidity and mortality globally; secondary damage mechanisms, particularly those occurring during the reperfusion process, significantly worsen the clinical prognosis [1]. IR syndrome was first described by Haimovici in 1960 as a serious complication observed after acute ischemic surgeries, and it has been shown that this condition is not limited to local tissues but can also cause systemic effects [2]. Ischemia–reperfusion injury (IRI) is a multifaceted, complex pathophysiological process characterized by a paradoxical increase in cellular damage and impairment of organ function following the restoration of blood flow after the ischemic period [3]. The release into the circulation of lactate, hypoxanthine, and other metabolites accumulated in the ischemic tissue during the reperfusion phase. Excessive production of reactive oxygen species (ROS) and the release of pro-inflammatory cytokines trigger a potent inflammatory response. This process often goes beyond ischemia-related cellular damage, leading to more widespread tissue destruction and microvascular dysfunction [4]. Therefore, IRI has been extensively studied in tissues with high metabolic demands, such as the heart, kidney, liver, brain, and skeletal muscle.

Lower extremity ischemia–reperfusion injury (LL-IRI) is a clinically significant peripheral vascular pathology. This condition, which can develop as a result of chronic arterial stenosis, thrombotic occlusion, trauma, atherosclerotic processes, or tourniquet application, particularly deepens tissue hypoxia with sudden arterial occlusion in acute embolic events due to atrial fibrillation [5]. With the onset of reperfusion, increased oxidative stress and inflammatory response are not limited to local muscle tissue but can spread to the systemic circulation, leading to secondary damage in distant organs such as the lungs, kidneys, and liver, and progressing to multiple organ failure syndrome [6,7].

One of the most critical consequences of ischemic stress at the cellular level is the disruption of calcium homeostasis. Decreased ATP production during ischemia impairs the ion pump function, leading to increased intracellular Ca^2+^ accumulation. Increased Ca^2+^ influx via voltage-gated calcium channels and receptor-mediated channels, combined with the deficiency of Na^+^/Ca^2+^ exchangers and Ca^2+^-ATPase pumps, dramatically increases cytoplasmic calcium levels. Excessive Ca^2+^ loading in mitochondria disrupts the electron transport chain, increases ROS production, and deepens oxidative stress [8]. Thus, a self-reinforcing cycle is formed between ion imbalance and oxidative damage. At this point, ion channels with high calcium permeability, especially TRPC6, are noteworthy. TRPC6: a non-selective cation channel expressed in brain, smooth muscle, kidney, and immune cells, exhibiting significantly higher permeability to Ca^2+^ than to Na^+^ [9]. Increased membrane depolarization and energy deficiency in an ischemic environment can affect TRPC6 activity and associated ion currents, increasing intracellular Ca^2+^ load. This, combined with secondary activation of voltage-gated calcium channels, can contribute to the exacerbation of contractile dysfunction and cellular damage in muscle tissue.

Peptide mediators involved in the regulation of energy metabolism are also important in IRI pathogenesis. Adropin is a secretory peptide encoded by the energy homeostasis-associated (ENHO) gene and involved in energy homeostasis [10]. In experimental models, adropin has been reported to reduce endothelial permeability under hypoxic and low-glucose conditions and to maintain blood–brain barrier integrity by modulating the Notch1 signaling pathway [11,12]. It has also been shown to increase perfusion and capillary density in a hindlimb ischemia model [13]. These findings suggest that adropin may limit ischemia-related tissue damage by supporting microvascular integrity.

Similarly, ISM-1 is an evolutionarily conserved glycoprotein that, while identified in developmental processes, also functions in metabolic regulation [14]. Glucose homeostasis is maintained by a balanced interaction between glucose uptake by peripheral tissues and hepatic and renal glucose production [15,16]. The ability of ISM-1 to lower glucose levels through insulin-independent mechanisms, suppress lipid synthesis, and increase protein synthesis makes it a potential link between energy metabolism and cellular stress response [17]. These regulatory effects on energy metabolism may be an important factor influencing cellular resilience during IR.

Another key regulator of the vascular response is prostacyclin (PGI_2_). PGI_2_, an arachidonic acid derivative eicosanoid, exhibits effects such as potent vasodilation, inhibition of platelet aggregation, and preservation of microcirculation. ILO, a synthetic, long-acting prostacyclin analog, induces vasodilation and suppresses platelet activation by activating prostacyclin receptors on vascular smooth muscle cells [18]. It is also reported to limit the inflammatory response by reducing leukocyte activation and pro-inflammatory mediator release [19,20]. Additionally, it has been shown to inhibit the production of endothelin-1, tumor necrosis factor (TNF-α), and adhesion molecules, and to reduce growth factors associated with fibrotic processes [21,22]. Studies have reported that iloprost preserves tissue integrity by reducing oxidative stress and inflammation in experimental skeletal muscle IR models [23,24,25].

When all these molecular and cellular data are evaluated together, it becomes clear that ion homeostasis, energy metabolism, and vascular regulatory mechanisms are closely interrelated in the pathogenesis of IR. Accordingly, the aim of the present study was to investigate the effects of ILO administration on gastrocnemius tissue using histopathological and biochemical parameters in an experimental IR model. Thus, the goal was to reveal the potential protective role of ILO on oxidative stress, inflammation, and cellular damage mechanisms through a holistic approach.

## 2. Materials and Methods

This study was conducted at the Experimental Research Center Unit of Firat University, Elazig, Turkey, following ethical committee approval dated 15 January 2020, number 2020/01, issued by the Firat University Animal Research Ethics Committee, Elazig, Turkey.

This study was designed, performed, and reported in strict accordance with the ARRIVE guidelines 2.0 (Animal Research: Reporting of In Vivo Experiments). All efforts were made to minimize animal suffering and reduce the number of animals used. The experimental design, sample size calculation, randomization, blinding, and statistical analysis plans were executed to ensure compliance with the Essential 10 checklist.

The rats used in our study were kept at 22–25 °C under a 12 h light (7:00–19:00) and 12 h dark (19:00–7:00) cycle. They were fed in special cages whose bottoms were cleaned daily. Feed was provided in stainless steel containers, and water in glass bottles (tap water). All rats were fed the same standard feed. Water and feed were provided ad libitum. The rats were housed in cages that were cleaned daily. Thirty male Wistar albino rats, aged 8–10 weeks, were divided into five groups:

Group I (Control group) (*n* = 6): No intervention was performed on the control group.

Group II (sham group) (*n* = 6): Rats in this group underwent laparotomy and abdominal aortic dissection. The stress and duration of the surgical procedure were equal for all five groups.

Group III (ILO group) (*n* = 6): Rats in this group were administered an intravenous infusion of 2 ng/kg/min ILO. ILO was administered intravenously at a dose of 2 ng/kg/min during reperfusion, in accordance with previous experimental IR studies reporting its protective effects on microcirculation, oxidative stress, inflammation, and skeletal muscle injury [23,24,25]. The infusion was initiated at the beginning of reperfusion because this period represents the critical phase in which oxidative stress, endothelial dysfunction, platelet activation, and leukocyte-mediated inflammatory injury are amplified.

Group IV (IR group) (*n* = 6): In this group of rats, the infrarenal abdominal aorta (IAA) was exposed by laparotomy performed through the midline of the abdomen in the supine position. The IAA was then clipped with a non-traumatic microvascular clamp for 120 min, after which the clips were released, reperfusion was allowed for 120 min, and the experiment was terminated.

Group V (IR + ILO group) (*n* = 6): Rats were immobilized in the supine position, and laparotomy was performed in the midline of the abdomen to dissect IAA. Later, IAA was clipped with a non-traumatic microvascular clamp for 120 min, and an ILO infusion of 2 ng/kg/minute was administered via the jugular vein after the clips were removed. The surgical procedure was completed after 120 min of reperfusion.

In our study, rats were anesthetized by intramuscular administration of 30 mg/kg ketamine hydrochloride (Ketalar, Pfizer, Groton, CT, USA) and 3 mg/kg xylazine hydrochloride 37 (Rompun; Bayer, Leverkusen, Germany).

Rats were anesthetized with additional doses of 1/3 of the initial dose as needed, ensuring spontaneous respiratory muscle function throughout the procedure. To prevent hypothermia, the operation was performed in a supine position under a heating lamp. The skin was aseptically prepared, and a laparotomy was performed in the midline of the abdomen. To maintain fluid balance, 10 mL of lukewarm saline was administered into the peritoneal space. The intestines were retracted to the left using wet gauze to access the abdominal aorta. Anticoagulation with 150 U/kg heparin (Nevparin, Mustafa Nevzat İlaç San., Istanbul, Turkey) was administered intravenously via the tail vein 2 min before aortic clamping. A lower extremity ischemia model was created in rats by clamping the IAA with a non-traumatic microvascular clamp. Absence of flow was confirmed distal to the clamp using a HADECO ES-101 EX handheld Doppler, Hadeco Inc., Kawasaki, Japan. The abdominal incision was kept closed to minimize heat and fluid loss. The induced ischemia was observed for 120 min. After occlusion, the abdomen was reopened, the microvascular clamp on the IAA was removed, and reperfusion was allowed for 120 min. A lower extremity ischemia model was developed by clamping the IAA with a non-traumatic microvascular clamp. Absence of distal perfusion during the time of aortic clamping and return of distal perfusion after release of the clamp were confirmed with handheld Doppler ultrasound.

### 2.1. Experimental Lower Extremity Ischemia–Reperfusion Model and Iloprost Administration

To study the lower extremity IRI, a model of lower extremity IR was established by clamping the IAA of rats for 120 min and then releasing the clamp for 120 min of reperfusion. This model of lower extremity skeletal muscle IR has been used in rat models of IRI in various organs and was shown to cause reproducible amounts of oxidative stress, inflammatory responses, and impairment of microcirculation. Consequently, damage to the various tissues of the lower extremity was induced in this model [4,6,23,24,25].

ILO was administered intravenously at a dose of 2 ng/kg/min by continuous infusion for the entire 120 min reperfusion period. This dose has been shown to improve endothelial function and to reduce IRI to skeletal muscle in various experimental models by decreasing oxidative stress, inflammation and by improving the local and peripheral microcirculation [18,19,20,21,22,23,24,25].

### 2.2. Sample Collection

At the end of the experiment, intracardiac blood was drawn from rats in all groups under anesthesia, and muscle tissue from the lower extremities was quickly removed. The collected blood samples were centrifuged at 4000 rpm for 5 min, and the resulting serums were stored at −80 °C until analysis.

Muscle tissue samples were fixed in 10% formaldehyde solution for histopathological and immunohistochemical studies. After fixation in 10% formaldehyde solution, they were washed with tap water. The washed tissues were subjected to a routine histological follow-up series. Subsequently, the tissues were embedded in paraffin blocks. Sections with a thickness of 4–6 µm were taken from these paraffin blocks onto polyzine-containing slides.

### 2.3. Biochemical Analysis

Serum TOS and TAS levels were determined by using ELISA kits for rats that were purchased from commercial market. The Rat Total Oxidant Status ELISA Kit (Shanghai YL Biotech Co., Ltd., Shanghai, China; Cat. No: YLA1392RA; Lot No: YL5886653758) was used for determining TOS levels. In addition, while the Rat Total Antioxidant Status ELISA Kit (Shanghai YL Biotech Co., Ltd., Shanghai, China; Cat. No: YLA1389RA; Lot No: YL2663863161) was used for determining TAS levels, the procedure was conducted by following the protocol for the ELISA kits, and all the reagents and serum samples were allowed to be brought to the room temperature prior to the measurements.

### 2.4. Immunohistochemical Study

Sections with a thickness of 4–6 µm thick were taken from paraffin blocks and placed on polylysine-coated slides. The deparaffinized tissues were passed through a graduated alcohol series and boiled in citrate buffer solution at pH 6 in a microwave oven (750 W) for 7 + 5 min for antigen retrieval. After boiling, the tissues were allowed to cool at room temperature for approximately 20 min, then washed with PBS (Phosphate-Buffered Saline, P4417, Sigma-Aldrich, St. Louis, MO, USA) for 3 × 5 min and incubated with hydrogen peroxide block solution (Hydrogen Peroxide Block, TA-125-HP, Lab Vision Corporation, Freemont, CA, USA) for 5 min to inhibit endogenous peroxidase activity. Tissues were washed with PBS for 3 × 5 min, then treated with Ultra V Block (TA–125-UB, Lab Vision Corporation, USA) solution for 5 min to prevent background staining. Afterward, immunohistochemical staining for ISM-1, adropin and TRPC6 was performed using the following primary antibodies: anti-ISM-1 (Elabscience Biotechnology Inc., Houston, TX, USA; Cat. No: E-AB-52045), anti-adropin (Abcam, Cambridge, UK; Cat. No: ab12800) and anti-TRPC6 (Lifespan Biosciences, Seattle, WA, USA; Cat. No: LS-C312900). Each of the primary antibodies were mixed with 1 drop of antibody diluent/PBS containing blocking solution to make a working solution at a 1:200 dilution ratio. The sections were incubated with the primary antibodies for 60 min at room temperature in a humidified chamber. Following primary antibody application, tissues were washed with PBS for 3 × 5 min and then incubated with a secondary antibody (biotinylated goat anti-polyvalent (anti-mouse/rabbit IgG), TP–125-BN, Lab Vision Corporation, USA) for 30 min in a humid environment at room temperature. After secondary antibody application, tissues were washed with PBS for 3 × 5 min, incubated with streptavidin peroxidase (TS–125-HR, Lab Vision Corporation, USA) for 30 min in a humid environment at room temperature, then transferred to PBS. Tissues were then treated with 3-amino-9-ethylcarbazole (AEC) Substrate + AEC Chromogen (AEC Substrate, TA-015 and HAS, AEC Chromogen, TA-002-HAC, Lab Vision Corporation, USA) solution. After obtaining an image signal under a light microscope, they were simultaneously washed with PBS. Tissues, which were counterstained with Mayer’s hematoxylin, were washed in PBS and distilled water, then covered with an appropriate capping solution (Large Volume Vision Mount, TA-125-UG, Lab Vision Corporation, USA).

The prepared specimens were examined and evaluated using a Leica DM500 microscope, Leica Microsystems GmbH, Wetzlar, Hessen, Germany and photographed (Leica DFC295, Leica Microsystems, Heerburg, Switzerland).

Histoscores were created based on the prevalence (0.1: <25%, 0.4: 26–50%, 0.6: 51–75%, 0.9: 76–100%) and severity (0: none, +0.5: very slight, +1: slight, +2: moderate, +3: severe) of immunoreactivity in the staining. Histoscore = prevalence × severity [26,27].

### 2.5. Statistical Analysis

Statistical analyses were performed using SPSS version 22.0 (IBM Corporation, Armonk, NY, USA) and GraphPad Prism (version 11). Prior to the study, sample size was determined using G*Power Software Version: 3.1.9.7 based on an effect size of f = 0.80, a statistical power of 85% (1 − β = 0.85), and a type I error rate of 5% (α = 0.05). Accordingly, a total of 30 animals were included in the study, with six animals allocated to each of the five experimental groups (N = 30; *n* = 6/group). The normality of data distribution was assessed using the Shapiro–Wilk test. Normally distributed data were analyzed using one-way analysis of variance (ANOVA), followed by Tukey’s multiple comparison test. Non-normally distributed data were analyzed using the Kruskal–Wallis test, followed by Dunn’s multiple comparison test for post hoc pairwise comparisons. Effect sizes were reported as eta squared (η^2^) for one-way ANOVA and eta squared based on the Kruskal–Wallis statistic [η^2^(H)] for the Kruskal–Wallis test, together with the corresponding *p* values. Normally distributed data are presented as mean ± standard deviation (SD), whereas non-normally distributed data are expressed as median (minimum–maximum). A *p* value < 0.05 was considered statistically significant.

## 3. Results

### 3.1. Biochemical Findings

#### 3.1.1. Serum Total Oxidant Status (TOS) Levels

**Figure 1:**
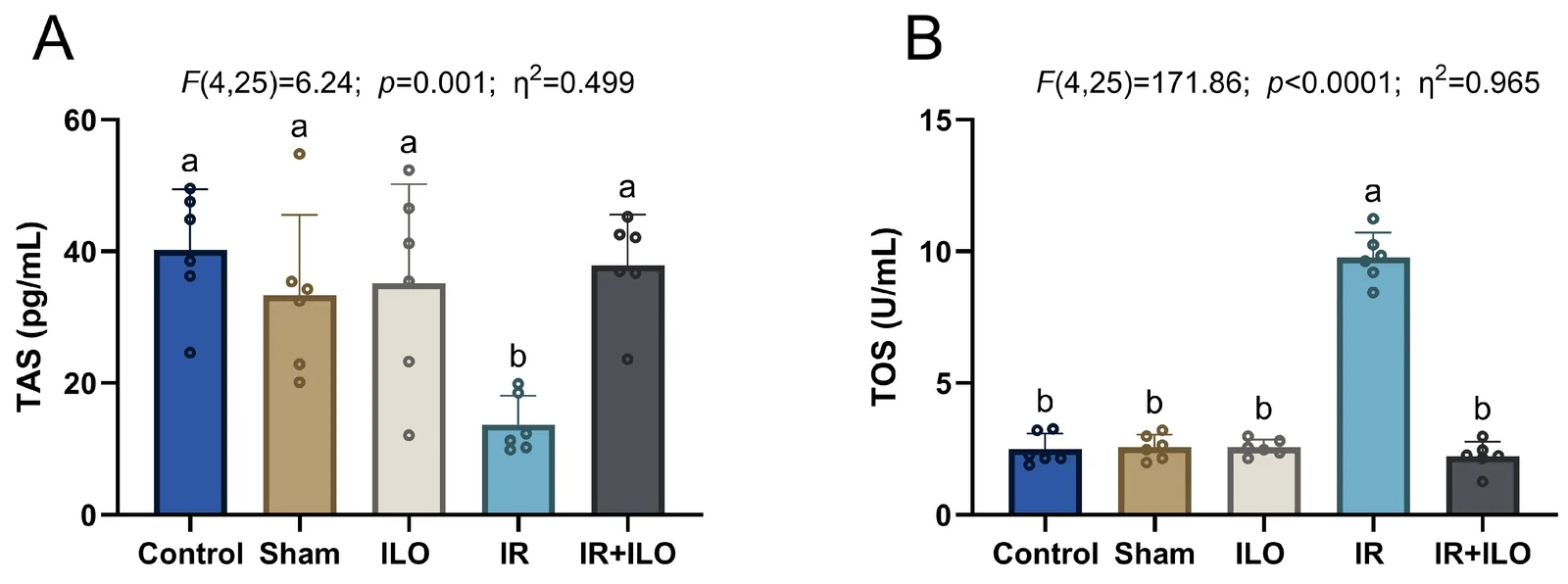
Effects of iloprost on serum total antioxidant status (TAS; Panel (**A**)) and total oxidant status (TOS; Panel (**B**)) in an experimental lower extremity ischemia–reperfusion injury model.

In the biochemical study evaluating serum TOS levels across all groups, compared with the control group, no statistically significant difference was observed in TOS levels in the Sham (*p* = 0.997) and ILO (*p* = 0.996) groups. In contrast, a statistically significant increase in TOS levels was observed in the IR group (*p* = 0.019). Compared with the IR group, the IR + ILO group showed a statistically significant decrease in TOS levels (*p* = 0.014) (Figure 1).

#### 3.1.2. Serum Total Antioxidant Status (TAS) Levels

In the biochemical study evaluating serum TAS levels across all groups, compared with the control group, no statistically significant difference was observed in TAS levels in the Sham (*p* = 0.997) and ILO (*p* = 0.998) groups. In contrast, a statistically significant decrease in TAS levels was observed in the IR group (*p* = 0.006). Compared with the IR group, the IR + ILO group showed a statistically significant increase in TAS levels (*p* = 0.021) (Figure 1).

Data showing normal distribution are presented as mean ± standard deviation and displayed as bar graphs. All individual data points are shown. Statistical analysis was performed using one-way analysis of variance, followed by Tukey’s multiple comparison test. Different lowercase letters above the groups indicate statistically significant differences; groups not sharing the same letter differ significantly (*p* < 0.05).

### 3.2. Immunohistochemical Findings

#### 3.2.1. TRPC6 Immunoreactivity

Light microscopy of immunohistochemical staining performed to test TRPC6 immunoreactivity revealed TRPC6 immunoreactivity in myocytes (black arrow) within muscle tissue.

**Figure 2:**
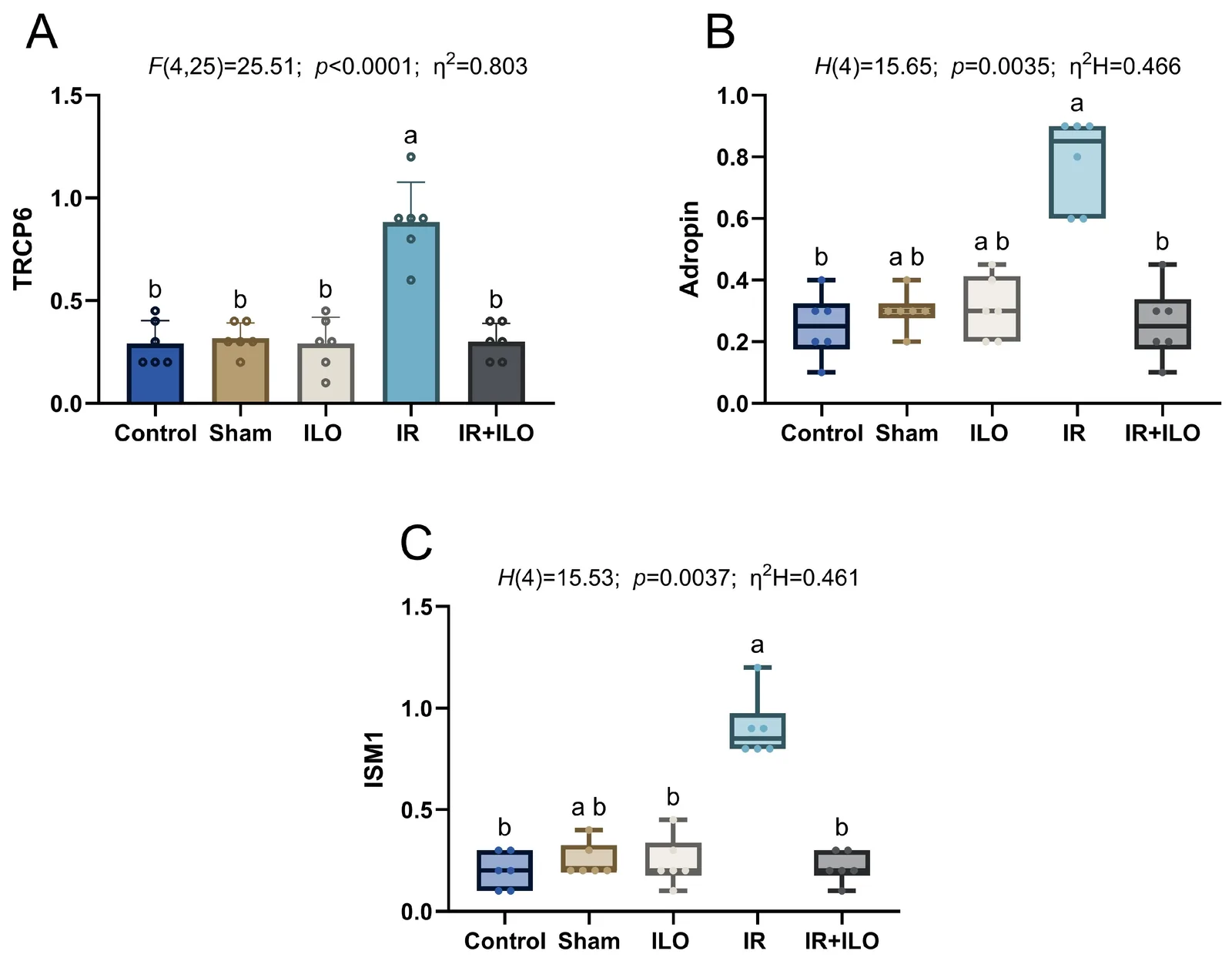
Effects of iloprost on transient receptor potential canonical 6 (TRPC6; Panel (**A**)), adropin (Panel (**B**)), and isthmin-1 (ISM1; Panel (**C**)) immunoreactivity histoscores in an experimental lower extremity ischemia–reperfusion injury model.

Compared with the control group, no statistically significant difference in TRPC6 immunoreactivity was observed in the Sham (*p* = 0.907) or ILO (*p* = 0.802) groups. In contrast, a statistically significant increase in TRPC6 immunoreactivity was observed in the IR group (*p* = 0.016). Compared to the IR group, a statistically significant decrease in TRPC6 immunoreactivity was observed in the IR + ILO group (*p* = 0.023) (Figure 2).

#### 3.2.2. Adropin Immunoreactivity

Light microscopy of immunohistochemical staining performed to test adropin immunoreactivity revealed that adropin immunoreactivity was observed in myocytes (black arrow) in muscle tissue.

Compared with the control group, no statistically significant difference in adropin immunoreactivity was observed in the Sham (*p* = 0.469) or ILO (*p* = 0.459) groups. In contrast, a statistically significant increase in adropin immunoreactivity was observed in the IR group (*p* = 0.006). Compared to the IR group, a statistically significant decrease in adropin immunoreactivity was observed in the IR + ILO group (*p* = 0.009). The findings are presented in Figure 2.

#### 3.2.3. ISM-1 Immunoreactivity

Light microscopy of immunohistochemical staining performed to test ISM-1 immunoreactivity revealed that ISM-1 immunoreactivity was observed in myocytes (black arrow) in muscle tissue.

Compared with the control group, no statistically significant difference in ISM-1 immunoreactivity was observed in the Sham (*p* = 0.506) or ILO (*p* = 0.670) groups. In contrast, the IR group showed a statistically significant increase in ISM-1 immunoreactivity (*p* = 0.007). Compared to the IR group, a statistically significant decrease in ISM-1 immunoreactivity was observed in the IR + ILO group (*p* = 0.017). The findings are presented in Figure 2.

Data showing normal distribution are presented as mean ± standard deviation and displayed as bar graphs, whereas data not showing normal distribution are presented as median (interquartile range), with whiskers representing the minimum and maximum values. All individual data points are shown. Normally distributed data were analyzed using one-way analysis of variance followed by Tukey’s multiple comparison test, while non-normally distributed data were analyzed using the Kruskal–Wallis test followed by Dunn’s multiple comparison test. Different lowercase letters indicate statistically significant differences; groups not sharing the same letter differ significantly (*p* < 0.05).

#### 3.2.4. Histological Findings

**Figure 3:**
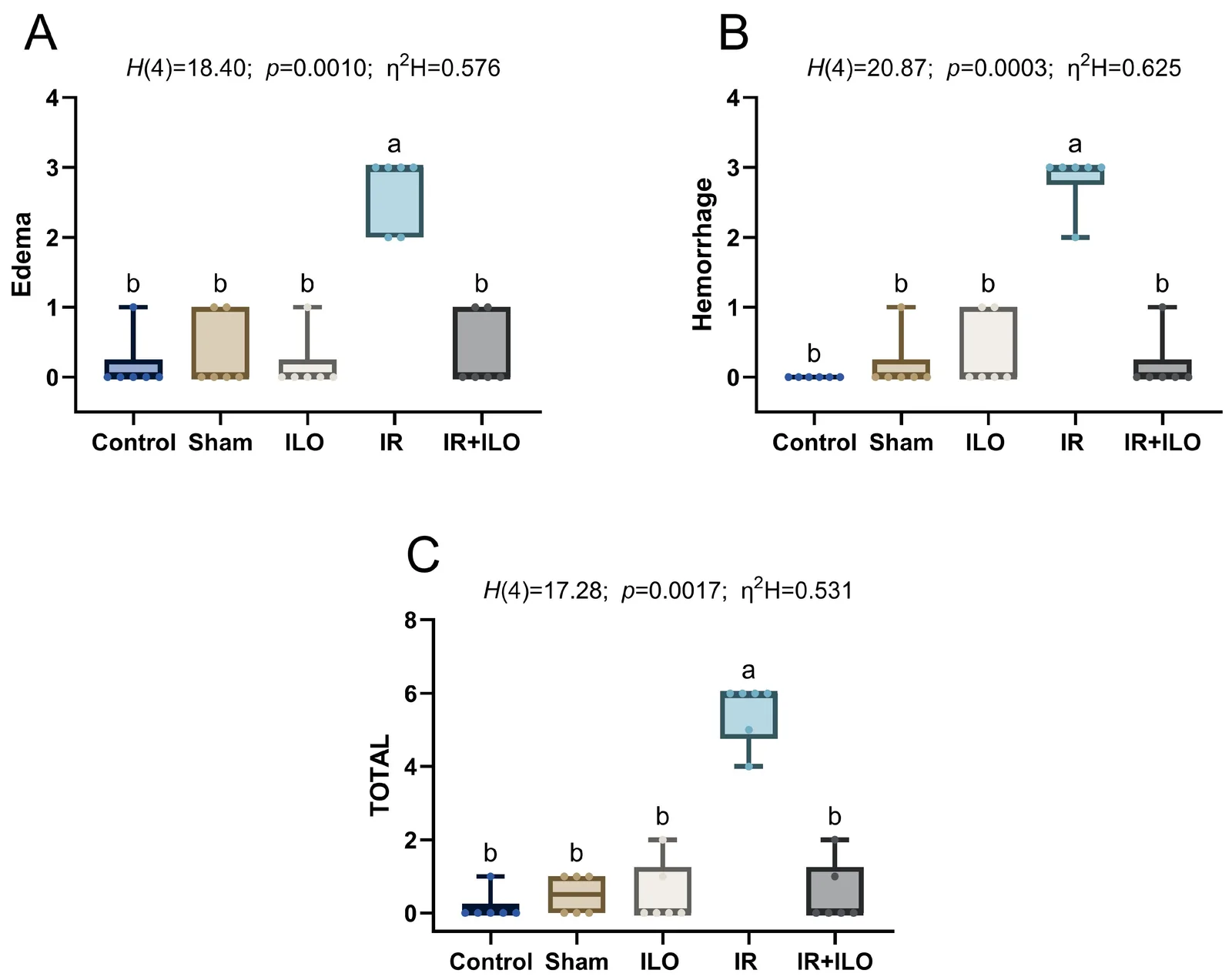
Effects of iloprost on histopathological edema (Panel (**A**)), hemorrhage (Panel (**B**)), and total histopathological injury score (Panel (**C**)) in an experimental lower extremity ischemia–reperfusion injury model.

As a result of light microscopy examination of muscle tissues stained with hematoxylin and eosin, all muscle tissues in the group were examined for edema and hemorrhage, and histopathological evaluation was performed for each animal (Figure 3). A histopathological evaluation score was created, and statistical analyses were performed.

**Figure 4:**
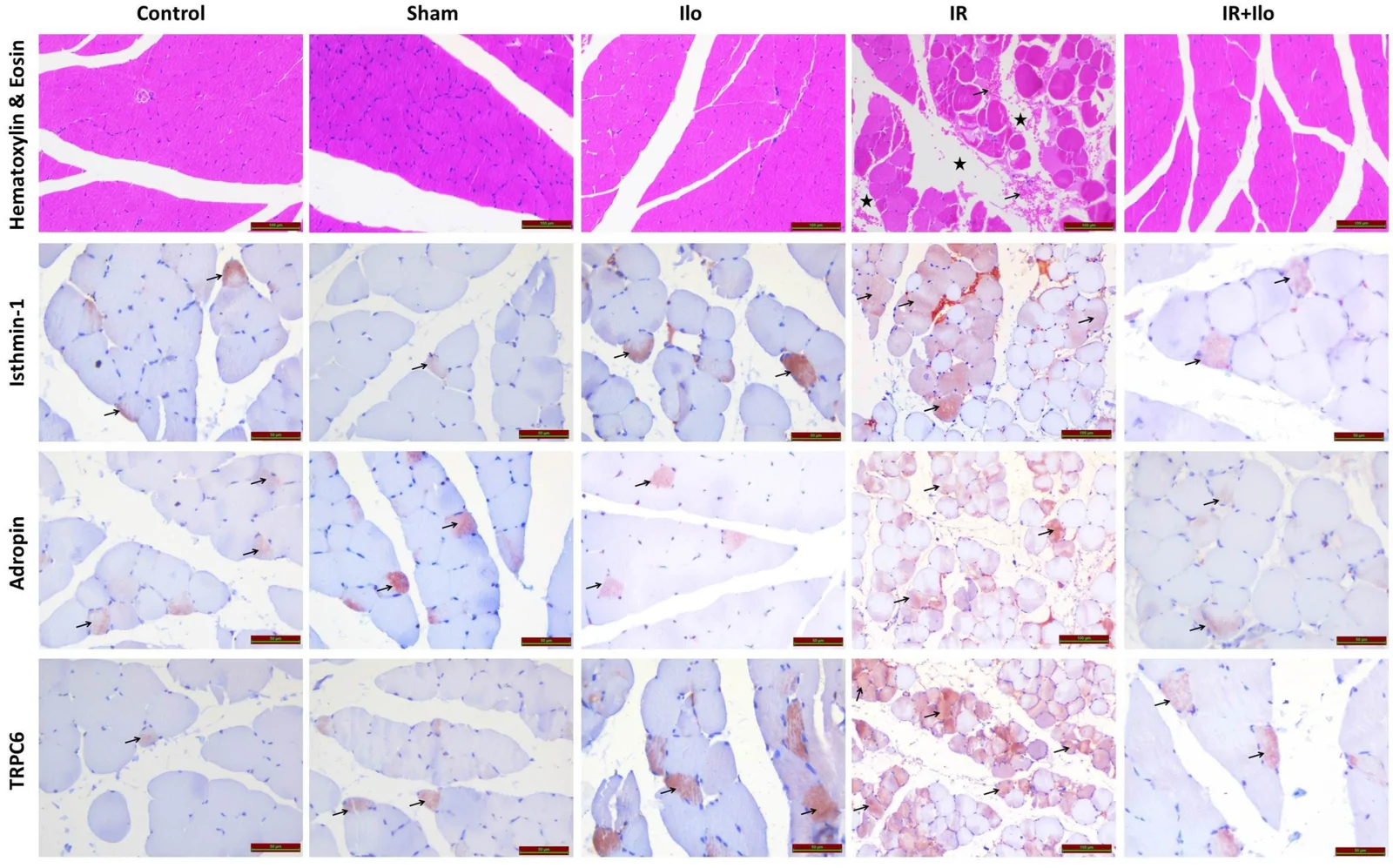
The effects of iloprost on Isthmin-1, adropin and TRPC6 immunoreactivities in control, sham, ILO and IR groups. Positive immunoreactivity is indicated by black arrows. Edema is indicated by stars. Scale bar: 50 µm; magnification: ×400.

Histopathological analysis of the paraffin-embedded skeletal muscle tissue sections that were stained with hematoxylin/eosin were performed for the assessment of edema and hemorrhage. The control, sham and ILO groups all showed well-preserved skeletal muscle fibers with low scores for edema and hemorrhage. The total histopathological score did not differ between the control and sham groups (*p* = 0.985), and between the control and ILO groups (*p* = 0.992). In the IR group, skeletal muscle in between the interstitial connective tissue showed the increased edema space and the hemorrhage areas. The total histopathological scores were significantly higher in the IR group compared to the control group (*p* = 0.002). The results indicated that the skeletal muscle in IR group showed the significant injury by the IR. In the IR + ILO group the scores for edema and for hemorrhage were both lower than in the IR group. The total histopathological score in the IR + ILO group was significantly lower than in the IR group (*p* = 0.016). Quantification of edema and of the areas of hemorrhage as well as a total score of the histopathological changes provided a complete assessment of the muscle injury. ILO in fact was not only able to reduce all components of the muscle damage but also their sum, evidently reducing the overall extent of IR-induced injury (Figure 3 and Figure 4).

Histopathological findings were evaluated in the control, sham-operated, iloprost-treated, ischemia–reperfusion, and ischemia–reperfusion-plus-iloprost groups. Data are presented as median (interquartile range), with whiskers representing the minimum and maximum values. All individual data points are shown. Statistical analysis was performed using the Kruskal–Wallis test followed by Dunn’s multiple comparison test. Different lowercase letters above the groups indicate statistically significant differences; groups not sharing the same letter differ significantly (*p* < 0.05).

## 4. Discussion

This study evaluated oxidative stress parameters, ion channel proteins, and the expression of energy metabolism-related mediators in muscle tissue injury induced by a lower extremity IR model. Serum TAS levels were significantly lower in the IR group than in the control group, and this reduction was partially reversed in the ILO-treated group. These findings demonstrate that depletion of antioxidant capacity is a key mechanism in IR injury and suggest that ILO may modulate the antioxidant defense system [28]. Similarly, increased oxidative stress and decreased antioxidant activity after IR have been demonstrated in many experimental models [29,30].

Serum TOS levels were significantly higher in the IR group than in the control group, and this increase was significantly reduced in the ILO group. These results indicate that increased ROS production and systemic oxidative load after reperfusion deepen damage in muscle tissue. PGI_2_ analogs have been reported in experimental models to reduce reperfusion injury through anti-inflammatory and antioxidant processes [31].

Immunohistochemical evaluation revealed a significant increase in TRPC6 immunoreactivity in the IR group. TRPC6 is a member of the channel family that regulates Ca^2+^ influx and has been shown in recent studies to contribute to calcium homeostasis disruption in IRI in skeletal muscle. Increased TRPC6 expression has been found to cause Ca^2+^ overload, apoptosis, and cell death signals [32].

In our study, the increased adropin immunoreactivity in the IR group may indicate a compensatory response aimed at maintaining energy balance and vascular integrity. Adropin has been suggested to support endothelial function and reduce cell permeability under hypoxic conditions, which can be interpreted as an adaptive response to reperfusion stress [11,12]. Although the number of direct IR studies involving adropin is limited, it has been shown in other tissues that energy metabolism-related signaling pathways affect post-ischemia damage, which supports the findings in our study [28].

ISM-1 is an evolutionarily conserved glycoprotein that plays a regulatory role in developmental processes and functions in the pathogenesis of cancer and inflammation through mechanisms such as apoptosis and anti-angiogenesis [17]. Its ability to increase protein synthesis and suppress lipid synthesis via the AKT–mTORC1–S6 pathway in liver and skeletal muscle makes it a key regulator of energy metabolism [33]. Furthermore, its effects in reducing oxidative stress and lipid peroxidation support its potential protective role in processes associated with metabolic diseases such as diabetes, obesity, and metabolic dysfunction-associated steatotic liver disease (MASLD) [34].

In this study, the increase in ISM-1 levels in IR-treated muscle tissue can be considered a compensatory response to increased oxidative and metabolic stress. The lower ISM-1 levels found in the ILO-treated group suggest that ILO reduces oxidative stress, thereby mitigating the metabolic stress response and reducing the need for ISM-1 expression. These findings indicate that ISM-1 may be a molecule associated with metabolic balance and cellular stress adaptation in IR pathogenesis. In addition, both TRPC6 and adropin immunoreactivity were reduced in the ILO-treated group compared with the IR group. This result suggests that ILO not only increases antioxidant capacity but may also regulate intracellular Ca^2+^ balance and cellular signaling pathways. Experimental model studies have also reported that ILO suppresses the inflammatory response and oxidative stress in IR-induced muscle damage [31]. The immunoreactivity for TRPC6, adropin and ISM-1 was increased in the IR group, and for these three molecules it was decreased in the IR + ILO group. Although the studied molecules are associated with different functions, their joint changes could reflect the complexity of the pathophysiological process of IRI. In fact, the IRI is a complex process including the production of reactive oxygen species, activation of inflammatory response, damage of endothelial cells and their dysfunction, disturbances of microcirculation and changes in the intracellular balance of ions [4,7,28,29].

The channel TRPC6 forms non-selective cation channels with a high Ca^2+^/squad ratio. Recently, these channels have been implicated in various processes that involve an increase in intracellular Ca^2+^-concentrations. In skeletal muscle, ischemia results in ATP-depletion and a collapse of the ion- and pH-homeostasis. Reperfusion subsequently is characterized by an increase in production of ROS and by a decrease in stability of cellular membranes. These events can induce an influx of Ca^2+^-ions via activation of TRPC6 channels, thus potentially inducing an intracellular Ca^2+^-overload that causes oxidative stress, mitochondrial dysfunction and apoptosis in skeletal muscle cells. The increased TRPC6 immunoreactivity observed in skeletal muscle fibers of the IR-group might therefore indicate an increased influx of Ca^2+^-ions via TRPC6 channels contributing to Ca^2+^-dependent cellular injury. These data are in line with recent evidence indicating a role for TRPC6 channels in apoptosis of skeletal muscle IRI. Adropin is a secreted peptide which is involved in energy homeostasis, and endothelial and vascular functions [10,13]. The increase in adropin immunoreactivity in the IR group of the present study may indicate an attempt to protect skeletal muscle from IR-induced endothelial dysfunction and impaired microcirculation. Previous studies have provided evidence that adropin can decrease endothelial permeability and protect the vascular barrier under conditions similar to ischemia, via activation of Notch1/Hes1 signaling [11,12]. The increased expression of adropin in the IR group of the present study was also found in experimental hindlimb ischemia and contributed to improved perfusion and capillary density in skeletal muscle [13].

ISM-1 is a secreted glycoprotein which is present in evolutionarily conserved form in animals from insects to vertebrates and is implicated in a variety of biological processes including metabolic regulation, inflammation, apoptosis and processes associated with angiogenesis [14,17]. More recently, ISM-1 has been reported to control glucose uptake and lipid metabolism in an insulin independent fashion [17] and it is likely that this molecule controls cellular metabolism via a number of different signaling pathways including the AKT–mTORC1–S6 pathway which is a major regulator of cell growth and metabolism [34]. ISM-1 has therefore been found to play a major role in controlling the metabolic activity of skeletal muscle, which is a highly metabolic tissue, and it is likely that in skeletal muscle ISM-1 plays a number of roles in controlling and coordinating a variety of different cellular and tissue functions. In the present study we have found that the ISM-1 immunoreactivity in tissue-resident memory T cells (TRM) from IR animals was increased when compared to TRM from non-ischemic animals, and it is likely that the increased ISM-1 immunoreactivity that was found in TRM from IR animals is indicative of the extensive metabolic reorganization in skeletal muscle which is attempting to overcome the severe energy-depleted state that exists in muscle as a result of ischemia. Thus, ISM-1 and other putative markers of injury would not only be expected to identify a number of different effects of IR injury, including the induction of apoptosis, the activation of inflammation and the initiation of repair by new vessel growth but also a number of effects that are associated with the attempt to compensate for and recover from these effects of IR injury. Indeed the finding that the immunoreactivity of the three injury associated/compensatory markers that were studied here, i.e., TRPC6, adropin and ISM-1, were all increased in the IR group of animals and that these increases were decreased by treatment of animals with ILO (a drug with a number of different biochemical and physiological actions including the action of a vasodilator, an anti-platelet agent, an anti-inflammatory agent and an agent which preserves the functional capability of the microcirculation) suggests that all of these markers are upregulated in skeletal muscle in response to IR, and that the changes in the TRM of animals treated with ILO, which have been shown here to be associated with a decrease in the extent of skeletal muscle injury which occurs as a result of IR [23,24,25,31], are associated with a reduction in the extent of calcium-dependent cellular stress and in the degree of compensatory metabolic reorganization in skeletal muscle which occurs as a result of IR.

Note that it is important to mention here again that no proof for a causal interaction between TRPC6, adropin and ISM-1 and their change of expression during skeletal muscle IRI in this study was given. In order to clarify the association found here for these three different markers a series of additional experiments like Western blots, RT-qPCR, ELISA on tissue homogenates, measurements of calcium influx into isolated cells, and the use of specific inhibitors for different signaling pathways have to be performed. Larger numbers of animals per group have to be included as well.

Overall, the biochemical and immunohistochemical data obtained reveal that increased oxidative stress, disruption of Ca^2+^ homeostasis, and the metabolic stress response in IR-damaged tissues are interrelated processes. The vicious cycle of increased ROS and Ca^2+^ overload deepens cellular damage, while mediators of energy metabolism, such as adropin and ISM-1, develop adaptive responses to this damage. ILO treatment appears to have a multifaceted protective effect by increasing antioxidant capacity, reducing oxidative load, and normalizing certain molecular expression profiles, particularly TRPC6.

In conclusion, these findings demonstrate that ILO is not only an agent that regulates vascular tone; it can also modulate redox balance, ion homeostasis, and metabolic signaling pathways. Further detailed molecular-level investigation of TRPC6, adropin, and ISM1-related signaling networks will contribute to the development of targeted therapy strategies for IRI.

## 5. Study Limitations

The present study has several limitations. For the first aspect, only three different genes/molecules (TRPC6, adropin, and ISM-1) have been investigated, so the complexity of the molecular network implicated in the IRI cannot be completely clarified. A second limitation of the present study is that the molecular evidence has been basically obtained by measuring some markers of serum oxidative stress and by immunohistochemical observations. Additional evidence have been obtained by Western blotting, RT-qPCR, and ELISA on tissue homogenates or by specific inhibition studies on different molecular pathways. A third limitation of the present study is that the used immunohistochemical method for the quantification of the expression of the different molecules of interest is a semi-quantitative one, thus it is subject to some bias due to observer subjectivity. To avoid this bias, sections have been blindly analyzed by different observers. However, more robust evidence will be obtained by future studies investigating larger numbers of animals and by using a more quantitative approach to study the molecules of interest. The last limitation is that an association between iloprost treatment and a decrease in the immunoreactivity for TRPC6, adropin, and ISM-1 has been found, but a direct causality between these molecules has not been proven.

## Data Availability

The data supporting the findings of this study are available from the corresponding author Ibrahim Murat Ozguler upon reasonable request.

## Abbreviations

The following abbreviations are used in this manuscript:

AEC	3 Amino-9-ethylcarbazole
ENHO	Energy homeostasis-associated
IAA	Infrarenal abdominal aorta
ILO	Iloprost
IR	Ischemia–reperfusion
IRI	Ischemia–reperfusion injury
ISM-1	Isthmin-1
LL	Lower extremity
PBS	Phosphate-buffered saline
PGI_2_	Prostacyclin
ROS	Reactive oxygen species

## References

[1] Temiz A., Yener A.U., Barutçu A., Gazi E., Altun B., Bekler A., Vural A., Özkan T., Kurt T., Erbag G. (2015). Are patients, who were previously diagnosed with coronary artery disease by coronary angiography, on optimal medical treatment?. Turk. Gogus Kalp Damar Cerrahisi Derg..

[2] Magnoni F., Pedrini L., Palumbo N., Cirelli M.R., Faggioli G.L. (1996). Ischemia: Reperfusion syndrome of the lower limbs. Int. Angiol..

[3] Cai S., Liu Y., Cheng Y., Yuan J., Fang J. (2022). Dexmedetomidine protects cardiomyocytes against hypoxia/reoxygenation injury via multiple mechanisms. J. Clin. Lab. Anal..

[4] Cearra I., Herrero de la Parte B., Moreno-Franco D.I., García-Alonso I. (2021). A reproducible method for biochemical, histological and functional assessment of the effects of ischaemia-reperfusion syndrome in the lower limbs. Sci. Rep..

[5] Eltzschig H.K., Eckle T. (2011). Ischemia and reperfusion--from mechanism to translation. Nat. Med..

[6] Cai J., Jiang Y., Zhang M., Zhao H., Li H., Li K., Zhang X., Qiao T. (2018). Protective effects of mitochondrion-targeted peptide SS-31 against hind limb ischemia-reperfusion injury. J. Physiol. Biochem..

[7] Zhang M., Liu Q., Meng H., Duan H., Liu X., Wu J., Gao F., Wang S., Tan R., Yuan J. (2024). Ischemia-reperfusion injury: Molecular mechanisms and therapeutic targets. Signal Transduct. Target. Ther..

[8] Ay Y.A., Yurdgulu E.E., Bayir Y., Halici Z. (2025). The role and behavior of voltage-gated calcium channels in ischemia/reperfusion. Cell. Signal..

[9] Dietrich A., Gudermann T. (2007). TRPC6. Transient Receptor Potential (TRP) Channels.

[10] Kumar K.G., Trevaskis J.L., Lam D.D., Sutton G.M., Koza R.A., Chouljenko V.N., Kousoulas K.G., Rogers P.M., Kesterson R.A., Thearle M. (2008). Identification of adropin as a secreted factor linking dietary macronutrient intake with energy homeostasis and lipid metabolism. Cell Metab..

[11] Yang C., DeMars K.M., Hawkins K.E., Candelario-Jalil E. (2016). Adropin reduces paracellular permeability of rat brain endothelial cells exposed to ischemia-like conditions. Peptides.

[12] Yu L., Lu Z., Burchell S., Nowrangi D., Manaenko A., Li X., Xu Y., Xu N., Tang J., Dai H. (2017). Adropin preserves the blood-brain barrier through a Notch1/Hes1 pathway after intracerebral hemorrhage in mice. J. Neurochem..

[13] Lovren F., Pan Y., Quan A., Singh K.K., Shukla P.C., Gupta M., Al-Omran M., Teoh H., Verma S. (2010). Adropin is a novel regulator of endothelial function. Circulation.

[14] Pera E.M., Kim J.I., Martinez S.L., Brechner M., Li S.Y., Wessely O., De Robertis E.M. (2002). Isthmin is a novel secreted protein expressed as part of the Fgf-8 synexpression group in the Xenopus midbrain-hindbrain organizer. Mech. Dev..

[15] Petersen M.C., Vatner D.F., Shulman G.I. (2017). Regulation of hepatic glucose metabolism in health and disease. Nat. Rev. Endocrinol..

[16] Samuel V.T., Shulman G.I. (2016). The pathogenesis of insulin resistance: Integrating signaling pathways and substrate flux. J. Clin. Investig..

[17] Menghuan L., Yang Y., Qianhe M., Na Z., Shicheng C., Bo C., XueJie Y.I. (2023). Advances in research of biological functions of Isthmin-1. J. Cell Commun. Signal..

[18] Olschewski H., Simonneau G., Galiè N., Higenbottam T., Naeije R., Rubin L.J., Nikkho S., Speich R., Hoeper M.M., Behr J. (2002). Inhaled iloprost for severe pulmonary hypertension. N. Engl. J. Med..

[19] Sahsivar M.O., Narin C., Kiyici A., Toy H., Ege E., Sarigül A. (2009). The effect of iloprost on renal dysfunction after renal I/R using cystatin C and beta2-microglobulin monitoring. Shock.

[20] Kiris I., Tekin I., Yilmaz N., Sutcu R., Karahan N., Ocal A. (2009). Iloprost downregulates expression of adhesion molecules and reduces renal injury induced by abdominal aortic ischemia-reperfusion. Ann. Vasc. Surg..

[21] Balbir-Gurman A., Braun-Moscovici Y., Livshitz V., Schapira D., Markovits D., Rozin A., Boikaner T., Nahir A.M. (2007). Antioxidant status after iloprost treatment in patients with Raynaud’s phenomenon secondary to systemic sclerosis. Clin. Rheumatol..

[22] Mittag M., Beckheinrich P., Haustein U.F. (2001). Systemic sclerosis-related Raynaud’s phenomenon: Effects of iloprost infusion therapy on serum cytokine, growth factor and soluble adhesion molecule levels. Acta Derm. Venereol..

[23] Tiryakioglu O., Erkoc K., Tunerir B., Uysal O., Altin H.F., Gunes T., Aydin S. (2015). The effect of iloprost and N-acetylcysteine on skeletal muscle injury in an acute aortic ischemia-reperfusion model: An experimental study. BioMed Res. Int..

[24] Bagis Z., Ozeren M., Buyukakilli B., Balli E., Yaman S., Yetkin D., Ovla D. (2018). Effect of iloprost on contractile impairment and mitochondrial degeneration in ischemia-reperfusion of skeletal muscle. Physiol. Int..

[25] Erer D., Özer A., Demirtaş H., Gönül İ.I., Kara H., Arpacı H., Çomu F.M., Oktar G.L., Arslan M., Küçük A. (2016). Effects of alprostadil and iloprost on renal, lung, and skeletal muscle injury following hindlimb ischemia-reperfusion injury in rats. Drug Des. Dev. Ther..

[26] Sayıt B., Atilgan R., Aslan M., Kuloğlu T., Özercan I.H., Hançer S., Yavuzkır Ş., Tepe B. (2026). Can Podocalyxin Immunoreactivity Facilitate Histopathological Diagnosis of Placenta Accreta Spectrum (PAS) Disorders?. J. Obstet. Gynaecol. Res..

[27] Özgüler M., Hançer S., Solmaz Ö.A., Kuloğlu T. (2025). Podocalyxin, Isthmin-1, and Pentraxin-3 Immunoreactivities as Emerging Immunohistochemical Markers of Fibrosis in Chronic Hepatitis B. Biomedicines.

[28] Zhou T., Prather E.R., Garrison D.E., Zuo L. (2018). Interplay between ROS and Antioxidants during Ischemia-Reperfusion Injuries in Cardiac and Skeletal Muscle. Int. J. Mol. Sci..

[29] Güler M.C., Tanyeli A., Ekinci Akdemir F.N., Eraslan E., Özbek Şebin S., Güzel Erdoğan D., Nacar T. (2022). An Overview of Ischemia-Reperfusion Injury: Review on Oxidative Stress and Inflammatory Response. Eurasian J. Med..

[30] Özer A., Koçak B., Sezen Ş.C., Arslan M., Kavutçu M. (2024). The Effect of “Proanthocyanidin” on Ischemia-Reperfusion Injury in Skeletal Muscles of Rats. Medicina.

[31] Avci T., Erer D., Kucuk A., Oztürk Y., Tosun M., Oktar G.L., Arslan M., Iriz E., Kavutcu M., Tatar T. (2014). The effects of iloprost on ischemia-reperfusion injury in skeletal muscles in a rodent model. J. Surg. Res..

[32] Xie D.G., Li J.H., Zhong Y.L., Han H., Zhang J.J., Zhang Z.Q., Li S.T. (2024). Role of TRPC6 in apoptosis of skeletal muscle ischemia/reperfusion injury. Cell. Signal..

[33] Jiang Z., Zhao M., Voilquin L., Jung Y., Aikio M.A., Sahai T., Dou F.Y., Roche A.M., Carcamo-Orive I., Knowles J.W. (2021). Isthmin-1 is an adipokine that promotes glucose uptake and improves glucose tolerance and hepatic steatosis. Cell Metab..

[34] Tchkonia T., Morbeck D.E., Von Zglinicki T., Van Deursen J., Lustgarten J., Scrable H., Khosla S., Jensen M.D., Kirkland J.L. (2010). Fat tissue, aging, and cellular senescence. Aging Cell..

